# Effects of a 6-week pelvic floor muscle training on neuromuscular activity in healthy young men: a pilot study

**DOI:** 10.3389/fpubh.2026.1781361

**Published:** 2026-04-10

**Authors:** Justyna Labun, Magdalena Piernicka, Barbara Sieńkowska, Anna Szumilewicz

**Affiliations:** Department of Physical Education, Gdansk University of Physical Education and Sport, Gdansk, Poland

**Keywords:** EMG, men’s health, pelvic floor muscles, pelvic health, surface electromyography

## Abstract

The pelvic floor muscles (PFM) play a crucial role in urinary continence and sexual function. Regular training of PFM has been extensively studied and is widely recommended to maintain a high quality of life, particularly among women. However, there is a notable lack of information regarding the effectiveness of PFM exercises in men. This study aimed to evaluate changes in neuromuscular activity of the PFM in men before and after a 6-week PFM training program. The exercises were performed independently by the participants and integrated into their regular strength-training routines as a short 5–10 min isolated PFM sessions. The study included 16 men aged 23 ± 4 years (M ± SD), who were healthy and did not report symptoms of pelvic floor dysfunction confirmed by the Incontinence Impact Questionnaire (IIQ-7), and the International Index of Erectile Function (IIEF-5). Neuromuscular activity of the PFM was assessed using surface electromyography (EMG) with anal probe (Lifecare Anal Probe PR-06), employing the TeleMyoTM2400 (DTS) system from NORAXON. The EMG assessment protocol included 11 voluntary PFM contractions followed by relaxations, based on the Glazer’s protocol. Immediately after the first EMG assessment, participants performed PFM contractions and relaxations using the biofeedback method. The same protocol was repeated after 6 weeks of training program. After the 6-weeks, a significant improvement was observed in all performed contractions. The mean amplitude of five 3-s quick contractions increased from 7.94 μV to 12.72 μV (*p* = 0.006; ES: *r* = 0.68). For five 10-s contractions, the mean value improved from 7.8 μV to 13.32 μV (*p* = 0.001; ES: *r* = 0.82). The 60-s hold contraction showed an increase from 6.39 μV to 10 μV (*p* = 0.001; ES: *r* = 0.82). Positive effects on neuromuscular activity of the PFM in men were observed after a 6-week training program. Regular performance of PFM exercises may be beneficial for maintaining the health and physiological functions associated with this muscle group. Therefore, such training should be widely recommended in men.

## 1 Introduction

The pelvic floor muscles (PFM) play a fundamental role in maintaining the health of the male urogenital system, particularly in urinary continence and sexual function (1). They provide structural support for the pelvic organs and contribute to the integrated function of the lumbopelvic stability (2). Proper strength, endurance, and coordination of PFM are essential for maintaining control over sphincter functions, including urinary and fecal continence (3). Pelvic floor dysfunctions (PFD), including urinary incontinence—defined as any involuntary leakage of urine (4)—is a very common condition with substantial economic, social, and psychological consequences. Nevertheless, these disorders remain frequently underdiagnosed and underestimated in the male population (5). Additionally, PFM are crucial for sexual functions. Erectile dysfunction (ED) refers to the consistent or recurrent inability to achieve and/or maintain a penile erection sufficient for satisfactory sexual intercourse (3). While ED is frequently associated with age-related conditions, its prevalence in men aged 40–70 years is reported to be 52%, increasing with age (3, 6). However, studies highlight a growing prevalence of ED, reaching up to 30% in men under 40 (7). This trend underscores the need to understand the phenomenon and identify effective treatment strategies for men. PFD often arises from a lack of activation, control, or muscle strength (8). In men, reduced muscle tone and altered contraction patterns have been linked to urinary incontinence (9) and may also directly affect erectile strength and the ejaculatory process.

Pelvic floor muscle training (PFMT) is a non-invasive, first-line treatment for urinary incontinence and PFD. While widely recognized for women’s health, this form of exercise remains underutilized among men. To date, the effectiveness of PFMT in men has been confirmed for the following: stress urinary incontinence after prostate surgery and, to a small extent in erectile dysfunction and ejaculation issues, including premature ejaculation (10–13). One of the most effective methods for teaching proper PFM exercise techniques is biofeedback (BF) with electromyography (EMG), which provides real-time visual feedback on the quality of PFM contractions (14, 15).

Our previous research conducted among nulliparous and pregnant women demonstrated the positive impact of PFMT combined with a single EMG BF session on the activation sequence of PFM (16–18). In this study, we focus on the effectiveness of PFMT in men, aiming to fill the gaps in data on this topic. The aim of our study is to evaluate whether a 6-week PFMT program, supported by a single BF session, can enhance the neuromuscular activity of the PFM in young, physically active, healthy men.

## 2 Materials and methods

This was a one-group experimental study involving 16 male volunteers (mean age = 23 ± 4 years).

The inclusion criteria were as follows: male sex, no diagnosed systemic or urogenital diseases, no history of abdominal or pelvic surgery, no signs of PFM dysfunction and regular participation in strength-training activity. The exclusion criteria included any current or past medical conditions that could affect pelvic floor function (e.g., neurological, urological, or proctological disorders), a history of surgical interventions in the pelvic or abdominal area, or any reported allergies or sensitivities to disinfectants, nickel, or other materials used in the laboratory. The characteristic of the study group is presented in Table 1. Before participating in the research, all participants provided written informed consent. The study was conducted at the Physical Effort Laboratory at the Gdansk University of Physical Education and Sport in Poland from October to December 2022 and was conducted in accordance with the principles of the Declaration of Helsinki and with the approval of the Bioethics Committee (KB-9/14, with subsequent amendments) at the Regional Medical Chamber in Gdansk.

**Table 1:** Characteristic of the study group.

Variable	Participants *n* = 16 Me (min; max)
Age	23 (19; 34)
BMI	28 (20; 33)
Incontinence Impact Questionnaire – Short Form	0 (0; 33.33)
International Index of Erectile Function*	24.5 (21; 25)

Values are presented as median and minimum and maximum values Me (min; max), BMI, body mass index; Incontinence Impact Questionnaire – Short Form (maximum score 100); International Index of Erectile Function (maximum score 25). *Data available for 12 participants; four participants were not sexually active and did not complete the questionnaire.

In the initial phase, participants completed questionnaire-based evaluations to establish baseline data and exclude individuals with potential PFD. To assess PFD, we used the Incontinence Impact Questionnaire – Short Form (IIQ-7) (19), which evaluates how urinary incontinence affects daily activities and quality of life. The IIQ-7 assesses four domains: physical, social, emotional, and occupational functioning. It consists of 7 items scored on a Likert scale from 0 (“not at all”) to 3 (“very much”). The final score is calculated as the mean of all responses and converted to a 0–100 point scale, where higher values indicate a greater impact of urinary incontinence on quality of life. The classification of the impact of urinary incontinence on quality of life follows the criteria proposed by Corcos et al. (20): a score of ≤50 points indicates good quality of life, a score between 50 and 70 points indicates moderate quality of life, and any score above 70 points indicates poor quality of life. For assessing male sexual function, we used the International Index of Erectile Function (IIEF-5) (21). Higher scores reflect better erectile function, while lower scores indicate more severe erectile dysfunction. Severity thresholds commonly used are: 22–25 = no erectile dysfunction, 17–21 = mild, 12–16 = mild-to-moderate, 8–11 = moderate, and 5–7 = severe. Additionally, participants responded to questions evaluating their knowledge and understanding of PFM exercises and their execution.

The EMG signals were recorded using the TeleMyo™ 2400 T (DTS) direct-transmission EMG system (NORAXON, Scottsdale, AZ, USA) in accordance with the SENIAM recommendations (22). To assess PFM activity, surface EMG was obtained using an anal electrode (Lifecare PR-06) that participants applied themselves in private. The raw EMG signals were sampled at 1,000 Hz and filtered at 6–500 Hz. Data were then full-wave rectified and smoothed using the manufacturer’s proprietary processing settings. For comparability between participants, all EMG amplitudes were normalized to the mean EMG value recorded during each participant’s baseline phase. For each contraction, the mean EMG amplitude was calculated over the active phase. Subsequently, the mean values from the five repetitions of each task type were averaged to obtain a representative EMG value for “quick flicks” and for “10-s contractions,” respectively. These averaged values were then used for statistical comparison between pre- and post-intervention measurements. All signal processing and normalization procedures were performed in the manufacturer’s software. Participants were examined during daytime hours and had not engaged in any training sessions earlier the same day. Readiness for testing was defined as being in good physical and mental condition, and participants were tested only when rested and free from marked psycho-emotional stress, ensuring they could comfortably complete the entire EMG assessment protocol. No specific recommendations were given regarding the timing or content of meals, or the timing of urination or defecation before the session.

Neuromuscular activity of the PFM was assessed using EMG and the Glazer’s protocol (23), a reliable and widely used method for evaluating PFM function. Isolated contractions were performed in response to automatically delivered “contract” and “relax” commands generated by the software, which minimized the risk of bias or interference from the examiner. Prior to the EMG assessments, participants were provided with all relevant instructions. Participants confirmed their understanding of the procedures, and all assessments were performed in a private and respectful environment, with the opportunity to ask questions at any stage. During the contraction phase, participants were instructed to perform a maximal voluntary PFM contraction, while during the relaxation phase, they were guided to fully release PFM muscles and focus on achieving complete relaxation. Additionally, participants were reminded not to hold their breath during the procedure. The sequence included: (1) an initial 10-s rest baseline, (2) five 3-s (quick) contractions each followed by 3-s of relaxation, (3) five 10-s contractions each followed by 10-s relaxations, (4) one 60-s contraction, and (5) the final 10-s relaxation phase. To ensure that participants learned and performed the correct PFM contraction technique, a BF sequence was also implemented. Two contraction and relaxation sequences were completed. The first was a “blind” sequence where participants could not see their muscle activity. Immediately afterwards, the second sequence was performed with BF, during which participants observed their PFM activity on a computer screen. Through BF, the aim was to enhance awareness of the contractions by helping participants learn to locate, isolate, and memorize the correct movement patterns. Participants reported no pain or discomfort throughout the assessment, and the measurement process was well tolerated by all participants.

After the initial assessment, participants performed PFM exercises independently for 6 weeks. The PFMT program was created based on the “Graduated Strength Training: A Pelvic Muscle Exercise Program” (37), a program developed at the University of Michigan, USA. It is structured around the gradual progression of exercise volume and intensity by increasing the duration of contractions, number of repetitions, and number of sets. This systematic progression was designed to improve PFM strength over time. Spanning 6 weeks, the program consists of three exercise sessions each week, lasting approximately 5–10 min per session. The program remains uniform for all participants, providing a consistent set of exercises without individual customization. The program was also designed to be easily integrated into daily routines, with recommendations to include PFM activation during training tasks. Participants were also educated about the importance of PFM, advised on healthy toileting habits and general recommendations for incorporating the exercises into daily practice. Alongside this program, participants continued their usual physical activities, which could include strength training and sports, supporting a holistic approach. For PFMT participants needed only a comfortable space, such as a mat for lying on their back, without the need for specialized equipment. A physiotherapist provided instructions for new exercises each week. The participants performed the exercises independently at home. They were also encouraged to maintain a training diary to track their exercise frequency, which serves as a mean of self-monitoring and reflection rather than compliance assessment. To enhance motivation, the instructor sent out weekly reminders to encourage regular engagement with the program. After the 6-week exercise intervention, we reassessed PFM function and collected updated questionnaire data using standardized measures.

Statistical analysis was performed using Statistica 13.1 software. Data distribution was assessed with the Shapiro–Wilk test, which confirmed that the variables did not follow a normal distribution. Therefore, descriptive statistics were expressed as medians with minimum and maximum values, and Interquartile range (IQR). To compare results before and after the intervention, the non-parametric Wilcoxon signed-rank test was applied, with the significance level set at *p* ≤ 0.05. Effect size (ES) was calculated based on *Z*-statistics using the formula *r* = *Z*/√*N*. The magnitude of effect was interpreted as small (0.1 < |*r*| < 0.3), medium (0.3 < |*r*| < 0.5), or large (|*r*| ≥ 0.5).

## 3 Results

We presented the characteristics of the study group in the Table 1. The participants were young, healthy men without PFD, such as urinary incontinence or sexual dysfunctions. The high BMI observed in the group may be attributed to the sport practiced by most participants—powerlifting—which is associated with increased muscle mass. The maximal voluntary contraction (MVC) of the PFM significantly improved. In terms of this value, we observed a 52% increase in muscle activity after intervention compared to measurements taken before the training intervention (*p* = 0.002; ES: *r* = 0.78). Before the intervention, the mean MVC value was 14.90 μV, whereas after the intervention, it was 22.60 μV. Due to a significant increase in MVC values, normalization to MVC was not performed, as this could have prevented the observation of the actual changes. Therefore, the analysis was first conducted on non-normalized data and then on data normalized to the rest baseline values, in order to preserve the observed change.

**Figure 1:**
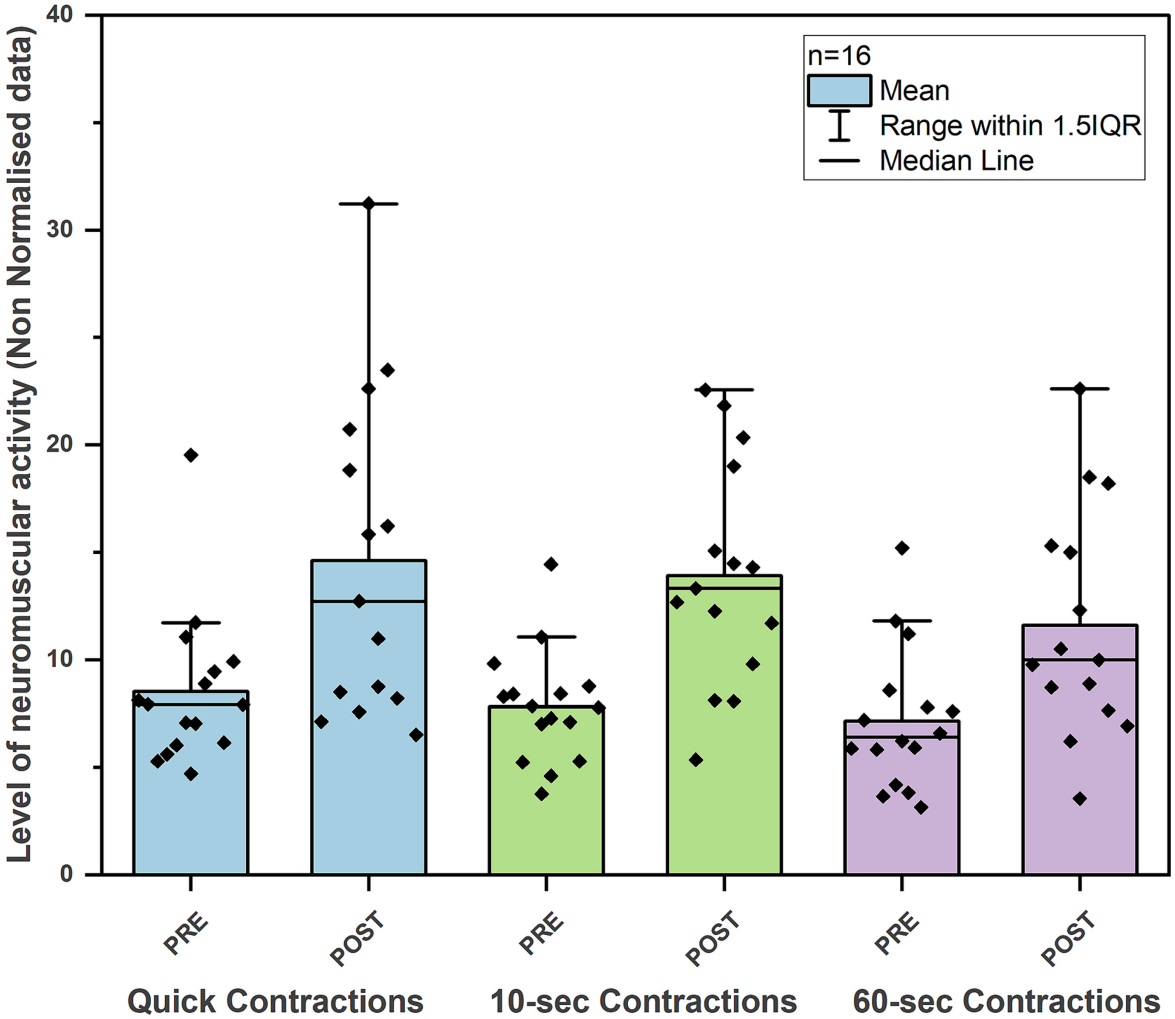
Changes in neuromuscular activity during quick flicks, 10- and 60-s pelvic floor muscle contractions before and after 6-week training program in young, active men (*n* = 16).

**Figure 2:**
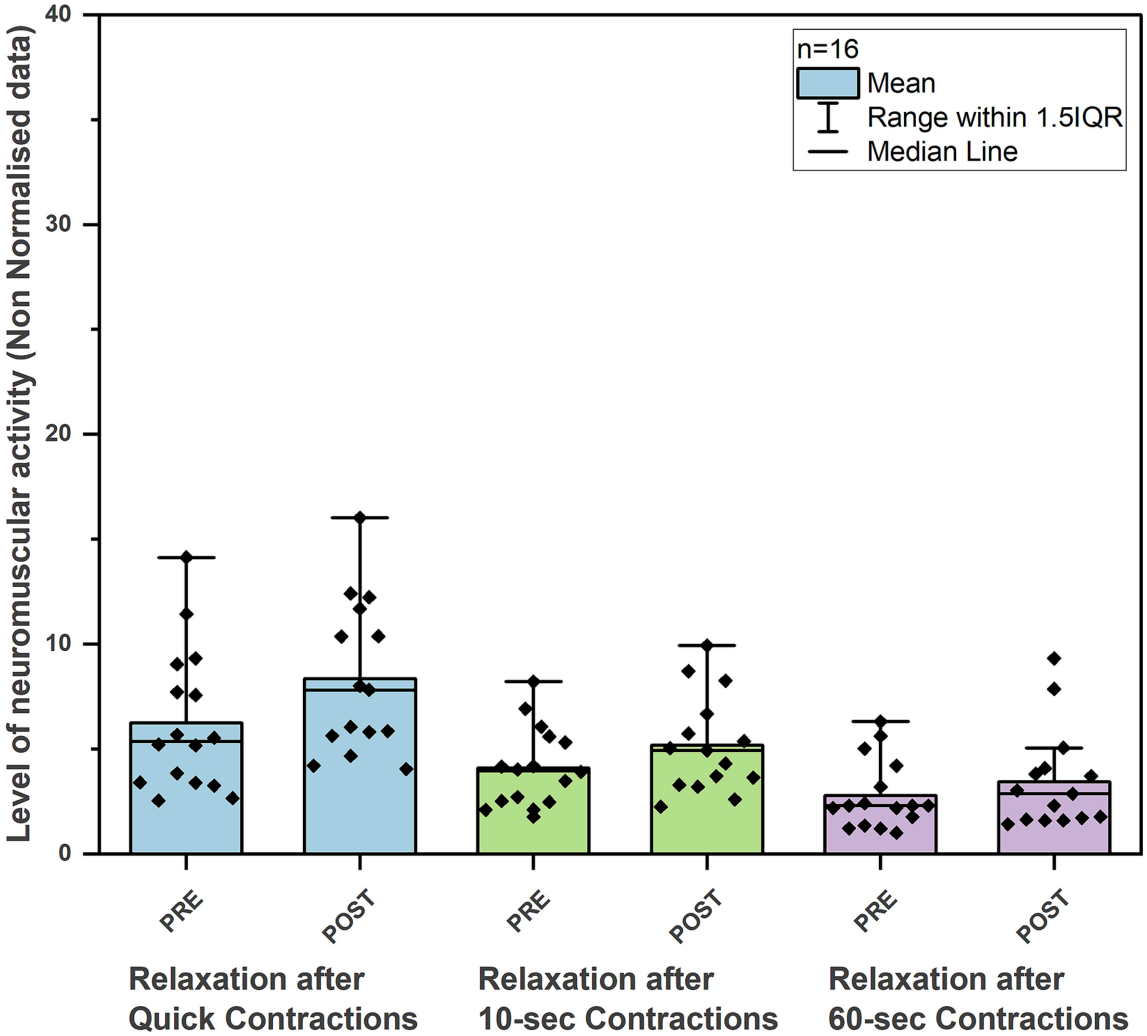
Changes in neuromuscular activity during relaxation after quick flicks, 10- and 60-s pelvic floor muscle contractions before and after 6-week training program in young, active men (*n* = 16).

In the non-normalized EMG data, we observed a significant improvement in all contractions performed after the intervention. Both quick contractions and longer holds (10-s and 60-s) showed significant improvement. The mean of five 3-s quick contractions increased from a mean EMG value of 7.94 μV to 12.72 μV (*p* = 0.006; ES: *r* = 0.68). The mean of five 10-s contractions improved from 7.8 μV to 13.32 μV (*p* = 0.001; ES: *r* = 0.82), and the 60-s hold contraction increased from 6.39 μV to 10 μV (*p* = 0.001; ES: *r* = 0.82) (Table 2 and Figure 1). The resting activity of the PFM either slightly increased or remained at the same level following the intervention. During the relaxations between quick 3-s contractions, we observed a greater increase in muscle activity compared to relaxations following longer contractions, from 5.52 μV to 7.81 μV (*p* = 0.013; ES: *r* = 0.61) (Table 2 and Figure 2).

**Table 2:** Level of PFM neuromuscular activity (EMG) value before and after training intervention (non-normalized).

Variables (μV)	EMG values before intervention Me (min–max); IQR	EMG values after intervention Me (min–max); IQR	Median change post-pre		ES	p* value
Maximal voluntary contraction (MVC)	14.90 (8.60–36.30); 5.6	22.60 (13.80–56.00); 17.5	+8		0.78	**0.002**
EMG activity at resting	4.04 (1.65–10.80); 2.94	5.05 (1.63–12.00); 3.48	+1		0.2	0.42
Mean of five quick 3-s contractions	7.94 (4.70–19.52); 3.79	12.72 (6.51–32.2); 12.51	+5		0.68	**0.006**
Mean of five relaxations between quick 3-s contractions	5.52 (2.54–14.12); 5.63	7.81 (4.04–16.02); 6.05	+2		0.61	**0.013**
Mean of five 10-s contractions	7.8 (3.75–14.44); 2.45	13.32 (5.34–22.56); 9.2	+5		0.82	**0.001**
Mean of five relaxations between 10-s contractions	3.96 (1,76–8.21); 2.96	4.39 (2.24–9.93); 3.39	0		0.45	0.06
60-s contraction	6.39 (3.14–15.20); 3.18	10.00 (3.54–22.60); 7.66	+4		0.82	**0.001**
Final relaxation	2.29 (1.0–6.30); 2.15	2.86 (1.42–9.32); 2.44	+1		0.31	0.21

PFM, pelvic floor muscles; Me, median; min, minimum; max, maximum; MVC, maximal voluntary contraction; IQR, interquartile range; ES, effect size calculated from *Z-*statistics using the formula *r* = *Z*/√*N* (small, 0.1 < ∣*r*∣ < 0.3; medium, 0.3 < ∣*r*∣ < 0.5; large, ∣*r*∣ ≥ 0.5). ^*^Wilcoxon signed-rank test. Significance level set at *p* ≤ 0.05. Bold values indicate statistically significant results.

For further analysis, the data were normalized to the rest baseline values. A statistically significant improvement occurred during exercises involving quick 3-s PFM contractions (quick flicks) (*p* = 0.03; ES: *r* = 0.53), as well as during 10-s contractions (*p* = 0.03; ES: *r* = 0.52). The final contraction, a 60-s PFM contraction, was not statistically significant, though we observed an improvement in activity, from an average of 173–306% (*p* = 0.07; ES: *r* = 0.45) (Table 3).

**Table 3:** Level of PFM neuromuscular activity before and after training intervention (EMG values normalized to baseline level).

Variables	EMG values before intervention % (min–max); IQR	EMG values after intervention % (min–max); IQR	Median change	ES	p* value
Average of five quick 3-s contractions	233% (91–670); 62.79	345% (195–614); 202	+112%	0.53	**0.03**
Average of five relaxations between quick 3-s contractions	155% (46–297); 27	200% (117–328); 90.4	+45%	0.41	0.1
Average of five 10-s contractions	206% (77–470); 105.7	361% (125–777); 235.7	+155%	0.52	**0.03**
Average of five relaxations between 10-s contractions	100% (46–126); 32	126% (67–215); 67.6	+26%	0.3	0.2
60-s contraction	173% (72–355); 80.1	306% (83–815); 231	+133%	0.45	0.07
Final relaxation	67% (18–106); 29.6	82% (29–190); 69.9	+15%	0.46	0.18

PFM, pelvic floor muscles; Me, median; min, minimum; max, maximum; IQR, interquartile range. Values were normalized and expressed as % of baseline EMG level. ES, effect size calculated from *Z-*statistics using the formula *r* = *Z*/√*N* (small, 0.1 < ∣*r*∣ < 0.3; medium, 0.3 < ∣*r*∣ < 0.5; large, ∣*r*∣ ≥ 0.5). *Wilcoxon signed-rank test. Significance level set at *p* ≤ 0.05. Bold values indicate statistically significant results.

After 6 weeks, all participants maintained normal PFM function related to continence or sexual function, as confirmed by questionnaire results. We observed no adverse effects of our intervention. Additionally, to better understand the study outcomes, we analyzed the participants’ knowledge about PFM prior to the experiment (Table 4). Nearly half of the participants (45%) were unaware of the location of the PFM, and more than half (55%) did not understand their importance for health. Furthermore, 69% of participants had never practiced PFM exercises before the study.

**Table 4:** Questionnaire of men’s knowledge about pelvic floor muscles before experiment.

Question	Answer (%)
Do you know where the pelvic floor muscles are located?	
Yes	55%
No	45%
Do you know the importance of pelvic floor muscle exercises for health?	
Yes	45%
No	55%
Have you practiced pelvic floor muscle exercises before?	
Yes, regularly	0
Yes, occasionally	12%
I tried the exercise once, but did not continue afterward.	19%
Never	69%
Have you ever performed the so-called “pee-stop” exercise?	
Yes, regularly	0
Yes, occasionally	0
I tried the exercise once, but did not continue afterward.	31%
Never	69%

## 4 Discussion

The most important finding from our study is the significant improvement in neuromuscular activity of the PFM across all contractions analyzed after the training program. There was a notable increase in the MVC value, as well as in both short (3-s) and longer (10- and 60-s) contractions. These results confirm the effectiveness of the intervention and the achievement of the research objective. MVC, as a measure of the maximal voluntary contraction generated by the muscles, plays a key role in assessing the muscles’ ability to generate force.

The effectiveness of PFMT in men is strongly supported by contemporary clinical studies. The best-documented clinical indication for performing PFMT is urinary incontinence following radical prostatectomy. Meta-analyses and systematic reviews have demonstrated that PFMT significantly accelerates the recovery of continence, particularly during the first months after surgery (24, 25). A systematic review including randomized controlled trials showed that both supervised and home-based PFMT substantially reduced post-prostatectomy incontinence and improved quality of life (26). Increasing evidence also points to the beneficial effects of PFMT on male sexual function, with randomized trials reporting improvements in erectile function following BF-assisted exercises (27, 28). Taken together, these findings indicate that PFMT is a safe and effective adjunctive therapy for a range of urological and sexual dysfunctions in men, although further standardization of therapeutic protocols is still required. In the case of men, knowledge about the role and benefits of PFMT is still limited, indicating a significant research gap, especially outside the context of rehabilitation after radical prostatectomy.

It is hypothesized that weakening of the PFM may decrease sexual satisfaction (29). In men, PFM contribute to penile rigidity by increasing intracavernosal and intraspongiosal pressure during muscle activation and also play a role in ejaculation (11). Promising results from studies by Lavoisier et al. (11) and Myers et al. (12) utilizing PFMT, have shown that such exercises can improve penile rigidity and alleviate or completely resolve erectile dysfunction. In men, reduced tension in PFM and changes in contraction patterns have so far been associated with urinary incontinence (9) but may also directly impact erectile strength and the ejaculation process. Issues related to impaired PFM relaxation have been observed in men with erectile dysfunction (ED). ED not only affects intimacy but also significantly lowers mood, self-esteem, interpersonal relationships, and overall life satisfaction (30). Research shows a strong correlation between erectile dysfunction and depression in men (31). Furthermore, sexual dysfunctions are often underreported and inadequately treated (32). Our study included healthy male participants without evident erectile dysfunction, as confirmed by the IIEF-5 questionnaire. Therefore, the above results cannot be fully interpreted in the context of treatment. Nevertheless, the improvement in EMG function appears promising as a potential preventive approach to PFD.

PFM play a key role not only in generating force but also in relaxation. Both of these aspects are important for the proper functioning of the urinary system, including processes such as micturition and defecation (33). Insufficient relaxation may disrupt the pelvic floor function, highlighting the importance of balanced PFMT that includes both contractions and relaxation. In our study, we observed a tendency toward difficulties with PFM relaxation following quick contractions. Elevated resting EMG activity may indicate delayed muscle relaxation after maximal exertion, particularly after the quick contraction assessments, where the time allotted for relaxation was intentionally limited. These findings are not interpreted as a negative effect of the training, but rather as a possible suggestion to extend the relaxation period in future protocols. Importantly, most participants demonstrated clinically normal relaxation levels, especially during the final relaxation phase. Impaired relaxation ability of the PFM has been observed in studies involving men with chronic pelvic pain syndrome (CPPS), who simultaneously experienced ED. The authors particularly emphasized the correlation between elevated resting tension and pain symptoms related to ED (34). In our study, no issues related to pelvic floor or erectile dysfunction were reported in the questionnaires, either before or after the 6-week exercise program, which may suggest the absence of adverse effects associated with the intervention.

All study participants were engaged in strength training, with most identifying as powerlifters. It has been hypothesized that individuals participating in strength training may be at increased risk of pelvic floor related issues. Specifically, some authors suggest that increased intra-abdominal pressure generated during high-load exercises may place additional demands on the PFM, potentially contributing to their overload and, consequently, weakening (35). However, this assumption has not yet been conclusively confirmed, particularly in the male population. Notably, strength training programs often do not include targeted PFM conditioning, which may further influence this potential risk. Further research is warranted to verify these hypotheses.

The effectiveness of PFM exercises has been widely documented in the literature. Other researchers indicate that the best results can be achieved by conducting training for at least 6–12 weeks (36). Varying the length of contractions, both short and maximal, as well as longer ones, aims to improve muscle strength and endurance, enabling targeted recruitment of fast- and slow-twitch fibers in the PFM (12). This is crucial not only for improving their function but also in the treatment and prevention of various disorders, such as urinary incontinence and sexual dysfunctions.

A particularly concerning finding is the low level of knowledge among men regarding PFM and their health implications. None of the participants had previously engaged in regular PFM exercises, and nearly half (45%) were unaware of the muscles’ location. Moreover, the majority (55%) lacked an understanding of their importance for overall health. This level of awareness may limit the possibilities for the prevention and treatment of PFD. These results highlight the need for educational initiatives that will help raise awareness among men about the function of these muscles and their importance for health.

Despite promising results, our study has its limitations. The lack of a control group and the relatively small sample size are significant limitations in interpreting the results. As this was a pilot study, our primary aim was to evaluate the feasibility of the protocol rather than to provide definitive comparative results. Including a control group would strengthen the design, but the current findings still offer valuable preliminary data for future research. We note that we did not standardize or record the time interval from the last micturition or defecation before testing, nor did we control for factors such as recent meals or sexual activity. Future studies should specify these preparatory conditions more precisely to further enhance the reproducibility of the protocol. Additionally, the absence of normative EMG reference values for healthy men limits the comparability of our results with other studies. This highlights the lack of international standardization for EMG outcomes in male pelvic floor assessment, which complicates the interpretation of observed changes and should be addressed in future research. It is also possible that a single BF session could have contributed substantially to the observed effects; however, the follow-up measurements were taken after 6 weeks, which suggests that the training effects were maintained over time. Nevertheless, despite the small sample, significant changes were observed, underscoring the potential for further research in this area. Our study included a specific cohort of physically active men engaged in strength training, which limits the generalizability of the findings to the broader male population. The questionnaire data on erectile function were based on 12 participants, as 4 individuals were not sexually active and therefore did not answer all questions; this should also be considered when interpreting the results. The 6-week training program is effective; however further research is needed to more accurately assess the long-term effectiveness of such interventions and their application in different age groups and with various dysfunctions.

## 5 Conclusion

The PFMT intervention supported by one BF session was associated with improvements in neuromuscular activity of the PFM in healthy, young men engaged in strength training. The results suggest that such exercises may contribute to improving pelvic floor neuromuscular activity, which could have a positive impact on men’s health. It is recommended to encourage men to regularly perform PFM exercises.

## Data Availability

The raw data supporting the conclusions of this article will be made available by the authors, without undue reservation.
